# Association Between Serum Biochemical Parameters and Kidney Stone Disease: An Exploratory Analysis of the 2017-2018 National Health and Nutrition Examination Survey (NHANES)

**DOI:** 10.7759/cureus.114628

**Published:** 2026-08-16

**Authors:** Ahmad R Abutalib, Mansour M Alnazari, Abdulaziz F Elyas, Rana M Alhejaili, Abdullah M Fallatah, Mawaddah O Alrehaili, Shouq K Alharbi, Asmaa S Aljohani, Zulfa W Kondarji, Mohammed B Mujlid

**Affiliations:** 1 College of Medicine and Surgery, Taibah University, Medina, SAU; 2 Department of Specialized Surgery, Taibah University, Medina, SAU; 3 Department of Clinical Nutrition, College of Applied Medical Sciences, Taibah University, Medina, SAU

**Keywords:** biochemical parameter, biochemical profile, kidney stone disease, nephrolithiasis, urolithiasis

## Abstract

Background: Kidney stone disease (KSD) is a multifactorial condition associated with individual and healthcare burden. Routine biochemical markers have yet to be fully explored for their association with KSD.

Methods: This is a retrospective analysis of cross-sectional data from the 2017-2018 National Health and Nutrition Examination Survey (NHANES). Those with a complete biochemical profile and kidney stone history data were included. Weighted multivariable regression analysis was utilized to adjust for confounding factors, and false discovery rate (FDR) correction was employed to account for multiple comparisons across biochemical markers.

Results: A total of 4,820 participants were included in our analysis, 477 of whom reported a history of kidney stones. The mean age was 49.48 ± 0.48 years. Serum bicarbonate was found to be significantly lower among those who reported a history of nephrolithiasis in comparison with those without in the unadjusted model (β = -0.320 mmol/L, P = 0.037), the model adjusted for age and sex (β = -0.301 mmol/L, P = 0.038), and the model adjusted for comorbidities (β = -0.340 mmol/L, P = 0.044). Following FDR, this association, however, did not remain significant across models (q = 0.296-0.352). Other biochemical parameters did not reach statistical significance for association with kidney stones in any model.

Conclusion: Serum bicarbonate levels demonstrated a modest, nominally significant inverse association with a history of nephrolithiasis, but this did not persist after correction for multiple comparisons and should be interpreted with caution given the cross-sectional design of this study. No other parameter showed a significant association with kidney stones. These findings, suggesting limited utility for single-time-point biochemical testing for stone risk assessment, should be considered exploratory and hypothesis-generating rather than conclusive evidence.

## Introduction

Kidney stone disease (KSD) is one of the most prevalent urological conditions in the United States (US), affecting approximately 1 in 11 adults [1]. Global data through 2021 show that despite the fact that the incidence and disability rates have declined over the past two decades, variability among regions, sociodemographic index (SDI) levels, and countries still persists [2]. These variations are associated with dietary habits and lifestyle factors, both of which contribute to the epidemiology of KSD [1]. Previous studies have reported that the risk of developing kidney stones is higher among older adults, men, and non-Hispanic White individuals [3].

KSD is understood to be a multifactorial, largely metabolic disease. Established risk factors identified in large prospective cohorts include obesity and weight gain [4], diabetes mellitus and associated insulin resistance [5], elevated body mass index in nationally representative samples [6], hypertension [7], and, more broadly, clustering of metabolic syndrome components [8]. Beyond these systemic associations, its pathogenesis at the renal level involves urinary supersaturation, crystal nucleation and aggregation, and deficiencies in stone inhibitors such as citrate [9].

Although urinary biochemical abnormalities are recognized as major risk factors for kidney stone formation, serum biochemical parameters are more frequently measured in clinical settings, and whether these more accessible parameters carry independent, population-level associations with kidney stone formation remains incompletely understood. Given this gap, our study aims to examine the independent association between routinely examined serum biochemical parameters and self-reported KSD in a nationally representative sample of US adults.

## Materials and methods

Data source

For the purpose of this study, data from the 2017-2018 National Health and Nutrition Examination Survey (NHANES were gathered. NHANES is a cross-sectional survey focusing on gathering a huge amount of information related to health and nutrition from non-institutionalized individuals in the US. All protocols of NHANES were approved by the Ethics Review Board of the National Center for Health Statistics (NCHS), and all participants have consented to the use of their data for research purposes [10]. Our analysis utilized the 2017-2018 NHANES cycle because it is the most recent cycle with the variables needed for this study with established sampling and weighting procedures. The study utilized data from a single cycle to maintain methodological consistency and avoid heterogeneity produced by changes in laboratory procedures and sampling methodology.

Study population

In this study, a publicly available two-year cycle (2017-2018) was utilized to conduct the analysis. Included individuals were those who underwent biochemical profile testing and had complete data regarding a history of diagnosis with kidney stones (n = 4,820). For each participant included in our study, data on completed biochemical profile, diagnosis of kidney stones, and possible confounding demographic and clinical covariates were extracted and aggregated. Weighting and variance estimation values were collected and included in the analysis to achieve nationally representative estimates.

Study variables

Kidney stones were the outcome of interest (dependent variable) in our analysis. It was collected by asking participants, “(Have you/your spouse/partner (SP)) ever had kidney stones?” An answer of "yes" was considered to be a positive history of kidney stones. As regards the exposure of interest (independent variable), we collected data on serum biochemical biomarkers. Biochemical parameters were measured on the Roche Cobas 6000 analyzer (c501 module; Roche Diagnostics, Basel, Switzerland) (refer to NHANES Laboratory Method Files for more details about the procedures and methods of sample analysis). Furthermore, repeat testing is randomly performed on 2% of all collected specimens by contract laboratories. Thus, we collected the following biochemical parameters as continuous variables: blood urea nitrogen (BUN) (mg/dL), serum creatinine (mg/dL), phosphorus (mg/dL), potassium (mmol/L), sodium (mmol/L), total calcium (mg/dL), bicarbonate (mmol/L), and serum uric acid (mg/dL).

Other covariates

Demographic covariates were collected through a trained interviewer using the Computer-Assisted Personal Interview (CAPI) system. Collected sociodemographic data included gender, age, educational level, marital status, and pregnancy status. Smoking status was also gathered from the Smoking-Cigarette Use questionnaire; those who answered “Every day” or “Some days” to the question “Do you smoke now?” were considered current smokers. Clinical covariates gathered included diabetes (from the Diabetes questionnaire), hypertension and hyperlipidemia (from the Blood Pressure and Cholesterol questionnaire), arthritis, gout, congestive heart failure, coronary artery disease (CAD), thyroid disease, and liver disease (from the Medical Conditions questionnaire). All previous comorbid conditions were gathered by a direct question, “Has a doctor or other health professional ever told (you/your SP) that (you/s/he) . . .had” followed by the respective chronic condition to be assessed.

Statistical analysis

All analyses accounted for the complex, multistage sampling design of NHANES by incorporating the two-year Mobile Examination Center sampling weight, masked pseudo-strata, and masked primary sampling units. Variance estimates were calculated using Taylor series linearization. Participants with an unknown kidney stone response or a missing/nonpositive examination weight were excluded from the analytic cohort. Continuous variables are presented as survey-weighted means with standard errors, whereas categorical variables are presented as unweighted frequencies with survey-weighted percentages. Group differences according to kidney stone history were evaluated using survey-adjusted tests. Missing questionnaire responses, including “don’t know,” “refused,” and structurally unanswered items, were excluded from the variable-specific denominator and were not recoded as negative responses. No imputation was performed.

Serum biomarker levels were compared between participants with and without a history of kidney stones using separate survey-weighted linear regression models for each biomarker. Participants without kidney stone history were used as the reference group; therefore, regression coefficients represented the mean biomarker difference between participants with and without kidney stone history. Model 1 was unadjusted. Model 2 was adjusted for age and sex. Model 3 was additionally adjusted for diabetes mellitus, hypertension, high cholesterol, and gout. Analyses were conducted using complete cases for the variables included in each model; consequently, the analytic sample varied across biomarkers and models. Results are reported as unstandardized regression coefficients, standard errors, 95% confidence intervals, and p-values. Because biomarkers were measured in different units, standardized regression coefficients (expressed in SD units) were additionally derived for visual comparison of relative effect sizes across biomarkers.

As a secondary analysis, survey-weighted logistic regression was used to examine the association between each serum biomarker and the odds of kidney stone history, with results expressed as odds ratios and 95% confidence intervals per one-unit increase in the corresponding biomarker. Statistical significance was defined as a two-sided p-value or false discovery rate (FDR)-adjusted q-value below 0.05, as applicable. All analyses were performed using Python 3.13.5 (Python Software Foundation, Fredericksburg, VA, USA).

## Results

Study participants characteristics

In our study, 4,820 individuals from the 2017-2018 NHANES cycle were included. These participants represent a survey-weighted sample of the adult population of the US. A total of 477 out of the 4,820 participants self-reported a history of kidney stones, whereas 4,343 reported no history of kidney stones. The weighted mean age of the included study population was 49.48 ± 0.48 years, with females constituting around 51.6% of the participants.

In regard to the demographic and clinical characteristics, they were categorized in accordance with kidney stone history. In terms of age, no significant difference was found between those with and those without a history of kidney stones (49.00 ± 0.92 vs. 49.97 ± 0.44 years, P = 0.34). Likewise, gender distribution was similar between both groups, with females constituting about 51.5% of the non-kidney stone group and 52.3% of the kidney stone group (P = 0.85).

Regarding comorbid conditions, a similar distribution was noted between the groups. Among the kidney stone group, diabetes mellitus was present in 14.5% of participants compared to 14.49% of those without a history of kidney stones (P = 0.79). Similarly, other comorbid conditions shared similar distribution among both groups with no significant difference between both groups in the prevalence of dyslipidemia (26.3% vs 26.9%, P = 0.29), hypertension (29.8% vs. 27.5%, P = 0.65), liver disease (5.02% vs. 5.0%, P = 0.80), thyroid disease (10.0% vs. 10.4%, P = 0.43), congestive heart failure (3.0% vs. 2.7%, P = 0.66), arthritis (29.5% vs. 29.4%, P = 0.63), or gout (5.4% vs. 5.7%, P = 0.71), and CAD (4.2% vs. 2.1%, P = 0.12) (see Table 1).

**Table 1:** Baseline characteristics of participants with kidney stone disease and no kidney stone disease Categorical variables were compared using the second-order Rao-Scott adjusted chi-square test, reported as an adjusted F statistic. Continuous variables were compared using survey-weighted general linear models, reported as an adjusted Wald F statistic. CAD: coronary artery disease

Characteristics	Total	No kidney stone disease	Kidney stone disease	Test statistics	p-value
Number (%)	4820	4343 (90.2)	477 (9.8)	-	-
Gender (Female, n (%))	2514 (51.6)	2269 (51.5)	245 (52.3)	0.037	0.85
Age (years, mean ± SE)	49.48 ± 0.48	49.97 ± 0.44	49.00 ± 0.92	0.829	0.34
Diabetes mellitus (Yes, n (%))	729 (14.49)	660 (14.49)	69 (14.5)	0.289	0.79
Hypertension (Yes, n (%))	1363 (29.5)	1230 (29.8)	133 (27.5)	0.462	0.65
Dyslipidemia (Yes, n (%))	1250 (26.4)	1126 (26.3)	124 (26.9)	1.309	0.29
Arthritis (Yes, n (%))	1398 (29.5)	1260 (29.5)	138 (29.4)	0.457	0.63
Gout (Yes, n (%))	280 (5.6)	250 (5.4)	30 (5.7)	0.402	0.71
Congestive heart failure (Yes, n (%))	160 (3.0)	146 (3.0)	14 (2.7)	0.480	0.66
CAD (Yes, n (%))	211 (4.0)	193 (4.2)	18 (2.1)	2.204	0.12
Thyroid disease (Yes, n (%))	541 (11.4)	490 (10.0)	51 (10.4)	0.939	0.43
Liver disease (Yes, n (%))	250 (5.03)	231 (5.02)	19 (5.0)	0.212	0.80

Serum biochemical parameters according to kidney stone history

The mean serum BUN concentration was found to be 14.87 ± 0.26 mg/dL, while the mean serum creatinine concentration was 0.88 ± 0.014 mg/dL. In regard to serum electrolyte concentrations, mean serum phosphorus, potassium, sodium, and total calcium were 3.59 ± 0.01 mg/dL, 4.08 ± 0.01 mmol/L, 140.27 ± 0.09 mmol/L, and 9.28 ± 0.02 mg/dL, respectively. Mean uric acid levels were 5.41 ± 0.05 mg/dL and mean bicarbonate levels were 25.57 ± 0.06 mmol/L.

When comparing serum levels of biochemical parameters between those with and without KSD, the mean serum bicarbonate levels were modestly lower among individuals with a history of kidney stones compared with those without (25.41 ± 0.11 vs. 25.73 ± 0.06 mmol/L), with a mean difference of -0.320 mmol/L. However, no statistically significant difference was noted for the remaining biochemical markers. Mean BUN concentrations, despite a non-significant difference, were lower among individuals with kidney stones compared with non-kidney stone individuals (14.69 ± 0.41 vs. 15.05 ± 0.21 mg/dL, P = 0.38). Likewise, when comparing those with and without a history of kidney stones, serum creatinine levels did not differ significantly between the two groups (0.87 ± 0.024 vs. 0.89 ± 0.011 mg/dL, P = 0.36). No significant difference was found for serum phosphorus concentrations (mean difference: 0.034 mg/dL, P = 0.42), potassium (mean difference: -0.018 mmol/l, P = 0.41), sodium (mean difference: -0.168 mmol/l, P = 0.37), total calcium (mean difference: -0.032 mg/dL, P = 0.25), or uric acid concentrations (mean difference: -0.091 mg/dL, P = 0.31) (see Table 2).

**Table 2:** Survey-weighted serum biomarker levels according to kidney stone history Serum biomarker levels were compared using survey-weighted general linear models, with the adjusted Wald F statistic reported. BUN: blood urea nitrogen

Characteristics	Total	No kidney stone disease	Kidney stone disease	Test statistic	p-value
Number (%)	4820	4343 (90.2)	477 (9.8)	-	-
BUN (mg/dL, mean ± SE)	14.87 ± 0.26	15.05 ± 0.21	14.69 ± 0.41	0.837	0.38
Creatinine (mg/dL, mean ± SE)	0.88 ± 0.014	0.89 ± 0.011	0.87 ± 0.024	0.898	0.36
Phosphorus (mg/dL, mean ± SE)	3.59 ± 0.01	3.57 ± 0.02	3.60 ± 0.03	0.677	0.42
Potassium (mmol/L, mean ± SE)	4.08 ± 0.01	4.09 ± 0.01	4.07 ± 0.02	0.697	0.41
Sodium (mmol/L, mean ± SE)	140.27 ± 0.09	140.35 ± 0.06	140.18 ± 0.17	0.850	0.37
Calcium (mg/dL, mean ± SE)	9.28 ± 0.02	9.29 ± 0.00	9.26 ± 0.03	1.414	0.25
Bicarbonate (mmol/L, mean ± SE)	25.57 ± 0.06	25.73 ± 0.06	25.41 ± 0.11	5.298	0.04
Uric acid (mg/dL, mean ± SE)	5.41 ± 0.05	5.46 ± 0.03	5.37 ± 0.09	1.111	0.31

Association between biochemical parameters and KSD

Regression analysis with sequential confounding factor adjustment was utilized to further assess the association between KSD and biochemical profile.

In the unadjusted analysis (model 1), a history of KSD showed a modestly significant association with lower serum bicarbonate levels compared to non-KSD (β = -0.320 mmol/L, P = 0.037). This association preserved its significance in the second model after adjusting for age and sex (β = -0.301 mmol/L, P = 0.038) and in the third model after adjusting for comorbid chronic conditions including diabetes mellitus, hypertension, high cholesterol, and gout (β = -0.340 mmol/L, P = 0.044).

As for the remaining biochemical parameters, no significant associations with kidney stones were observed in all three regression analysis models. Though non-significant, BUN has demonstrated an inverse association in the unadjusted model (β = -0.370 mg/dL, P = 0.371), which was attenuated after adjusting for confounders, demonstrating no significant difference between the kidney stones and non-kidney stones group (β = 0.108 mg/dL, P = 0.784). Similarly, creatinine in serum was consistently not associated with KSD in all three models (regression coefficients ranging from -0.020 to -0.032 mg/dL).

**Figure 1:**
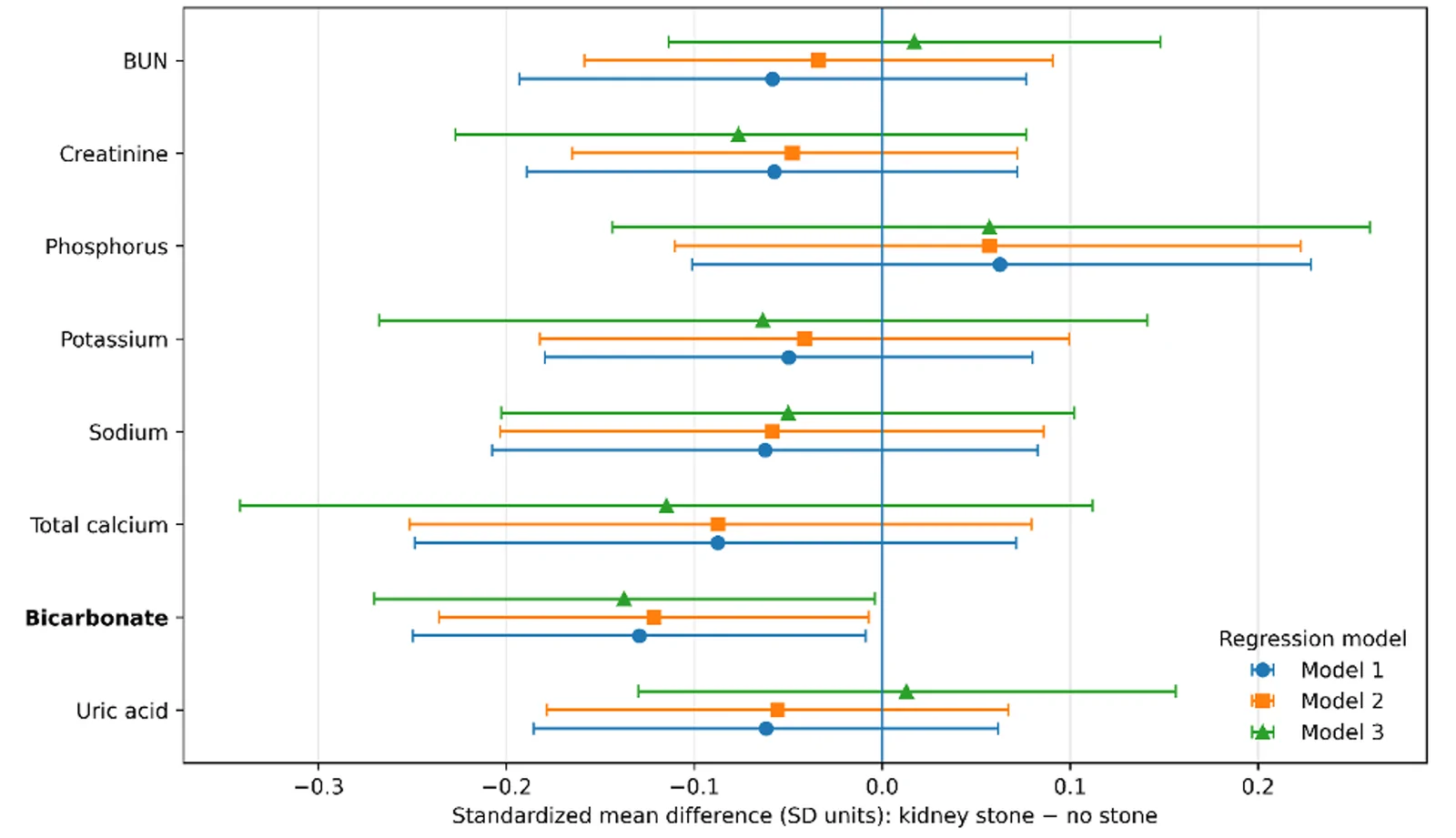
Survey-weighted standardized differences in serum biomarker levels according to kidney stone history Complex survey-weighted linear regression with Taylor series linearization. Points represent survey-weighted standardized regression coefficients, and horizontal lines represent 95% confidence intervals. Negative coefficients indicate lower serum biomarker levels among participants with a history of kidney stones compared with those without. Model 1 was unadjusted; Model 2 was adjusted for age and sex; and Model 3 was adjusted for age, sex, diabetes, hypertension, high cholesterol, and gout. Serum bicarbonate showed nominal significance across all three models, although no association remained statistically significant after false-discovery-rate correction. Bold text indicates statistical significance. BUN: blood urea nitrogen

**Figure 2:**
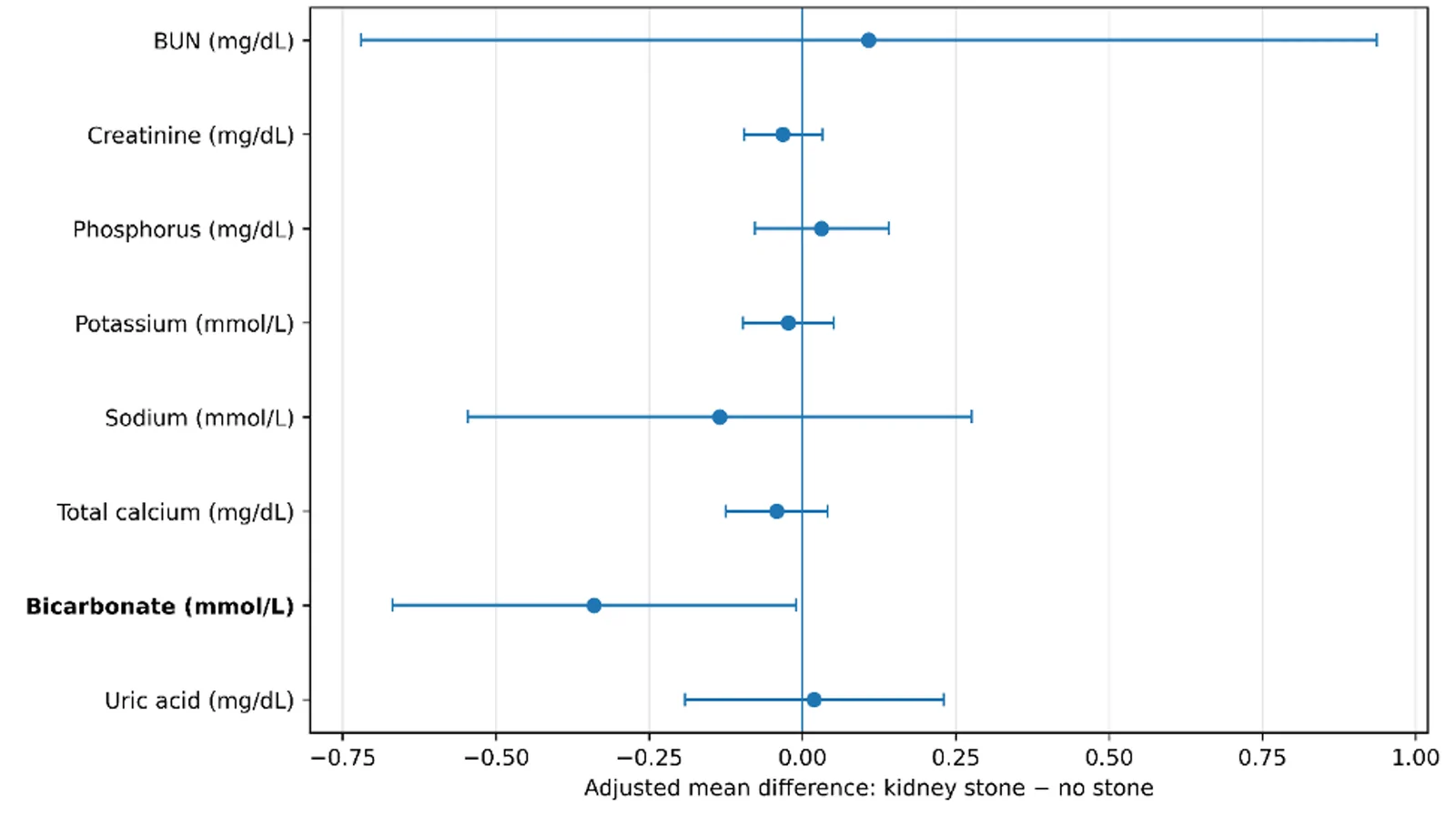
Available-covariate adjusted differences in serum biomarkers (unstandardized) Model 3 adjusted for age, sex, diabetes, hypertension, high cholesterol, and gout. Values represent unstandardized mean differences in native biomarker units (matching the β coefficients reported in the text). Because biomarkers are measured in different units, coefficient magnitudes should not be compared directly across markers. No association remained significant after FDR correction. Bold text indicates statistical significance. BUN: blood urea nitrogen; FDR: false discovery rate

Furthermore, serum potassium and sodium demonstrated negative coefficients in all three models; however, these differences were modest and statistically non-significant after adjustment (all P > 0.40). Likewise, total calcium levels showed small, statistically non-significant inverse associations with KSD (β ranging from -0.032 to -0.042). Serum phosphorus, despite demonstrating a small positive coefficient in each model, did not reach statistical significance. Uric acid levels were lower among those with a history of kidney stones after adjusting for age and sex, but its effect diminished following adjustment for additional comorbidities, resulting in a near-null estimate after full adjustment (β = 0.191 mg/dL, P = 0.849). Figure 1 demonstrated standardized differences across all three models, and Figure 2 shows the corresponding unstandardized adjusted mean differences from model 3.

FDR was conducted for each model to account for multiple comparisons. Following FDR, none of the included serum biochemical parameters remained statistically significant. Despite the fact that serum bicarbonate showed nominal statistical significance in all three regression analysis models before correction, the FDR-adjusted q values were 0.296, 0.308, and 0.352 for models 1, 2, and 3, respectively.

## Discussion

In our study, we analyzed cross-sectional data from 4,820 NHANES 2017-2018 participants, focusing on assessing the relationship between biochemical markers and a self-reported history of KSD. Following the analysis, serum bicarbonate showed a nominally modest inverse association with KSD in all three models. However, this association did not stand after multiple comparison correction and should be interpreted as directionally suggestive rather than a confirmed observation. On the contrary, other biochemical parameters (including BUN, creatinine, phosphorus, potassium, sodium, total calcium, and uric acid) did not demonstrate a significant association in any of the models.

Our finding in regard to the marginal association between bicarbonate levels and KSD is biologically plausible, and it is consistent with previously published studies in the literature, which often link acid-base status to stone formation. Even modest reductions in serum bicarbonate cause a considerable degree of systemic acid load, which subsequently results in proximal tubular citrate reabsorption, thereby lowering urinary citrate, a vital inhibitor of calcium stone formation, while also acidifying the urine, which facilitates uric acid and calcium oxalate crystal precipitation [9]. This link has been more clearly elaborated in chronic kidney disease patients, as a published retrospective cohort study over a multi-year follow-up concluded that lower bicarbonate levels and a trending-down bicarbonate pattern were associated with higher occurrence rates of kidney stones [11]. Not accounting for underlying tubular acidification disorders in our single time-point measurement likely masked a valid underlying signal, which might give explanation to why the association between bicarbonate levels and kidney stones lost its significance after FDR correction, despite being directionally consistent with these prior work findings.

As for uric acid, despite being regarded as an essential factor contributing to lithogenesis, it lacked significance in all models. Current evidence regards uric acid as a passive component in the formation of pure uric acid stones (where urinary aciduria is the main driver), an active component in calcium oxalate crystallization in hyperuricosuric calcium stone formation, and as a major constituent in ammonium urate stones [12]. Notably, in all of the previously mentioned mechanisms, the risk of lithogenesis was mainly determined by urinary uric acid concentration and urine pH, not serum uric acid levels, as serum uric acid is a relatively unreliable surrogate for the urinary handling of urate. This likely explains the null association found in our study for serum levels of uric acid, total calcium, phosphorus, and other electrolytes, where renal handling affects supersaturation and crystallization in the urinary tract more than serum concentrations.

Likewise, the absence of significant association between the kidney stone and non-kidney stone groups is consistent with the direction, but not the timing, of the body of literature on kidney stones and renal function. Studies often found that a history of kidney stones is associated with increased risk of chronic kidney disease and end-stage renal disease in the future [13,14]. This association was supported further by a meta-analysis of more than 4.7 million participants [15]. However, these studies describe the trends in renal function after kidney stones, often years of follow-up, rather than a single point-in-time analysis.

In our study, findings regarding the non-significant association between chronic comorbid conditions such as diabetes, hypertension, and high cholesterol levels and a history of kidney stones contrast with the body of literature identifying these as risk factors for kidney stone formation [4-8]. These are likely due to study design differences, as our study represents a single-time-point analysis for those with and without a history of kidney stones rather than following individuals across many years, which subjects the study to reverse causation, risk factor normalization, and survivor-type biases. This also stands true for the observation of no significant association between CAD and kidney stone history, as the literature repeatedly reported higher cardiovascular risk among individuals with a history of nephrolithiasis [16,17], a case in which cross-sectionally designed studies cannot reliably establish temporality.

Several strengths support the validity of these findings, including the use of a large, nationally representative sample with standardized laboratory methodology and complex survey weighting to generate estimates applicable to the broader US adult population. The application of sequential adjustment models and FDR correction also provides a conservative, transparent approach to managing the multiple-comparisons problem inherent in testing several biochemical markers simultaneously, reducing the likelihood of reporting spurious associations.

However, several limitations warrant consideration. First, the cross-sectional design precludes causal inference and is susceptible to reverse causation, particularly given that self-reporting of kidney stone history may reflect events occurring years before biochemical sampling, this is particularly true for the bicarbonate finding, as those with a history of kidney stones may have received subsequent clinical education on citrate or alkali supplementation, which may alter the levels of bicarbonate independent of the pathophysiology of lithogenesis itself. Second, data on kidney stones and comorbidities are self-reported, which is subject to recall and misclassification bias, and does not capture the full picture, including stone composition, recurrence, timing of stone events relative to serum sampling, or the presence of underlying metabolic or tubular disorders that might modify the observed biochemical associations. In addition to the previous two pitfalls, serum biochemical parameters were measured at a single time point and not over 24 hours through urinary excretion, which is far more accurate proximal to stone formation than serum values. Also, while FDR correction reduces the risk of false-positive findings, it may correspondingly increase the risk of type II error, potentially obscuring true but modest associations, such as that observed for bicarbonate. Additionally, our analysis was confined to the 2017-2018 NHANES cycle only; while this preserves consistency in the methodology and laboratory procedures, it might limit the statistical power when compared to pooled, multiple-cycle analysis; thus, the null findings observed in our studies should be confirmed through larger samples before being considered conclusive. Moreover, our multivariable models did not account for dietary intake such as total fluid intake, sodium, and animal protein consumption, which might be a source of unmeasured confounding that should be included in future analyses. Finally, the findings of this study may have been underpowered by the relatively small number of individuals reporting kidney stones, masking small true associations, and the null findings observed in our study should not be interpreted as definitive evidence of absence of association.

## Conclusions

In this exploratory analysis of NHANES 2017-2018 data, only bicarbonate showed a directionally consistent, nominally significant inverse association with a history of nephrolithiasis, and this association did not survive correction for multiple comparisons. No other routinely measured parameter demonstrated a significant relationship with kidney stone history in any model. These observations indicate that isolated single-time-point biochemical testing might be of limited value in assessing the risk for kidney stones. These findings, however, should be considered as hypothesis-driving rather than definitive evidence.
